# Lymph node metastasis related gene BICC1 promotes tumor progression by promoting EMT and immune infiltration in pancreatic cancer

**DOI:** 10.1186/s12920-023-01696-4

**Published:** 2023-10-25

**Authors:** Feilong Meng, Shuai Hua, Xuedong Chen, Nanfeng Meng, Ting Lan

**Affiliations:** 1 Minimally invasive Center of Hepatobiliary and Pancreatic surgery, The Second Hospital of Harbin, Harbin, Heilongjiang China; 2 Hepatobiliary and Pancreatic Surgery, The Second Hospital of Harbin, Harbin, Heilongjiang China; 3 Department of Hepatopancreatobiliary Surgery, The Second Affiliated Hospital of Harbin Medical University, Harbin, Heilongjiang China; 4 Department of Rehabilitation, The Second Hospital of Harbin, Ward A, Harbin, Heilongjiang China

**Keywords:** Pancreatic cancer, BICC1, Lymph node Metastasis, EMT, Immune microenvironment, Prognosis

## Abstract

**Background:**

Pancreatic cancer (PC) is one of the most aggressive abdominal malignancies with a poor prognosis and it is urgent to find effective biomarkers for prediction. Although BICC1 expression is related to the survival, no evidence for its role in PC development has been found.

**Methods:**

We used RNA-seq data to screen for molecular markers highly associated with lymph node metastasis. The Cancer Genome Atlas (TCGA) and International Cancer Genome Consortium (ICGC) public databases were used to analyze the expression and prognosis of Differential Expressed Genes (DEGs) in PC. R studio was used for visualization and functional analysis.

**Results:**

BicC Family RNA Binding Protein 1 (BICC1) was a lymph node metastasis-related DEGs in PC patients. Our study found that BICC1 mRNA levels in the tumor tissue were significantly higher and associated with poorer prognosis. Enrichment analysis found that BICC1 was enriched primarily in the Epithelial Mesenchymal Transition (EMT) pathway. Using the ESTIMATE and CIBERSORT algorithms, we found that BICC1 was related to immune cell infiltration. As a regulator of multiple immune checkpoints, BICC1 was also involved in PC’s immune response.

**Conclusions:**

BICC1 has the potential to be a new marker in association with lymph node metastasis as well as immune infiltration of PC. In addition to being a prognostic indicator, it may also be a potential therapeutic target.

## Introduction

One of the most aggressive forms of malignant cancer, pancreatic cancer (PC) accounts for the third largest number of cancer-related deaths [1]. Around 48,220 persons died of PC worldwide in 2021, making for 7.9% of all cancer fatalities [1]. The treatment of PC has progressed and is now widely used, including surgical resection, immunotherapy, radiotherapy, chemotherapy, etc. Because PC is aggressive, resistant to drugs, and hard to detect early, the survival rate is only 8–10% after 5 years [2, 3]. Lymph node metastasis affects the effectiveness of treatment in addition to being a key factor in the development of PC [4, 5]. Therefore, there is a need to identify an effective biomarker to assess lymph node metastasis and distinguish high-risk patients earlier. Moreover, targeted therapy based on gene mutation and regulation of pancreatic tumor microenvironment has been confirmed to bring survival benefits to PC patients [6]. However, the molecular regulatory mechanism targeting lymph node metastasis of PC has not been fully studied. We hope to explore prognostic markers related to lymph node metastasis of PC, so as to provide more ideas for targeted therapy of PC.

Bicaudal-C (BICC) consists of tandem repeats of Heterogeneous Nuclear Ribonucleoprotein K homology (KH) and KH-like (KHL) domains located at the N-terminus, separated from the C-terminal Sterile alpha motif (SAM) domain by a serine-glycine-rich sequence [7]. The KH region allows BICC to bind to “AU” enriched sequences in mRNA’s 3’ untranslated regions (UTR), which controls the stability of the mRNA [8, 9]. BICC Family RNA Binding Protein 1 (BICC1) as a Protein Coding gene has been found to play essential roles in human physiology and pathology. BICC1 is a genetic determinant of osteoblastogenesis and polycystic kidney disease [10, 11]. In addition, BICC1 negatively regulates Wnt signaling and assists embryonic development by regulating the gene expression [12]. BICC1 has been implicated in gastric cancer progression and invasion, and correlates with immune infiltrates [13]. In addition, some studies also found that BICC1’s aberrant expression contributes to the development of other malignant tumors: oral cancer, Wilms tumor and non-small cell lung cancer [14, 15]. However, He et al. [16] only found that BICC1 is a prognosis related gene of pancreatic ductal adenocarcinoma. How BICC1 promotes PC the occurrence, development, and associated biological behavior remains uncertain.

In this study, we first tried to explore whether lymph node metastasis related gene BICC1 can predict the prognosis of PC and its relationship with immune cell infiltration and immune checkpoint. Furthermore, we use bioinformatics analysis to try to prove that BICC1 provides a potential therapeutic target for PC immunotherapy and can be used as a biomarker for further research.

## Materials and methods

### Data

From the TCGA database (https://portal.gdc.cancer.gov/) we downloaded RNA-seq data from PC and normal pancreatic tissue [17]. In the TCGA database, which contains 179 PC tissue samples of 178 PC patients and 4 normal pancreas samples, clinical pathology data were collected, such as stage, grade, and survival time. From the University of California Santa Cruz (UCSC) database (https://xenabrowser.net/) was downloaded the TCGA TARGET Genotype-Tissue Expression (GTEx) dataset [18]. Our study also collected clinicopathological data and RNA-seq data in order to assess the quality of BICC1 expression in predicting the prognosis of PC from the International Cancer Genome Consortium (ICGC) database which contains 234 PC patients (http://dcc.icgc.org/) [19] As a validation dataset, the ICGC database was used. We obtained the protein expression and related clinicopathological data of BICC1 using the University of Alabama at Birmingham Cancer (UALCAN) database (http://ualcan.path.uab.edu/index.html) [20]. For data management, R software was used.

### Identification and Functional Enrichment analysis of DEGs

The “Limma” package of R software was used to identify DEGs between 48 PC patients with N0 stage disease and 123 PC patients with N1 stage disease in the TCGA database [21]. In the analysis of DEGs, a P-value < 0.05 was selected based on | log2 (fold change)| > 1.5. In the TCGA database, genes associated with overall survival (OS) were defined as a p value < 0.05 through Cox regression analysis. We used OS as a prognostic factor, which was defined as the time from surgery to death. We named these genes OS related genes (OSRGs). ClusterProfiler R package was used for the enrichment analysis, including gene set enrichment analysis (GSEA), Gene Ontology (GO) analysis, and Kyoto Encyclopedia of Genes and Genomes (KEGG) analysis [22].

### Construction and validation of clinical prognostic models

The TCGA database provided all information regarding PC patients. A total of 178 PC patients were classified into groups based on BICC1 expression levels. The median expression of BICC1 is used as the cutoff value. Table 1 shows demographic and clinical data about patients. Kaplan-Meier plots were generated to examine the correlation between OS and the survival rate, and ROC curves were generated to evaluate the accuracy of the model’s predictions over time. Predictive indicators were identified using both univariate and multivariate Cox proportional risk regression analyses. In order to create the nomogram and plot calibration curves, the ‘rms’ package and the survival package were used.

**Table 1:** Relationship between BICC1 mRNA expression and clinical features of PC in TCGA database

Characteristic	Low expression of BICC1	High expression of BICC1	*P*-value
n	89	89	
T stage, n (%)			0.672
T1	5 (2.8%)	2 (1.1%)	
T2	12 (6.8%)	12 (6.8%)	
T3	69 (39.2%)	73 (41.5%)	
T4	2 (1.1%)	1 (0.6%)	
N stage, n (%)			**0.006**
N0	33 (19.1%)	17 (9.8%)	
N1	51 (29.5%)	72 (41.6%)	
Pathologic stage, n (%)			0.320
Stage I	14 (8%)	7 (4%)	
Stage II	69 (39.4%)	77 (44%)	
Stage III	2 (1.1%)	1 (0.6%)	
Stage IV	3 (1.7%)	2 (1.1%)	
Gender, n (%)			0.175
Female	35 (19.7%)	45 (25.3%)	
Male	54 (30.3%)	44 (24.7%)	
Age, n (%)			**0.036**
≤ 65	39 (21.9%)	54 (30.3%)	
> 65	50 (28.1%)	35 (19.7%)	
Histologic grade, n (%)			0.369
G1	19 (10.8%)	12 (6.8%)	
G2	47 (26.7%)	48 (27.3%)	
G3	20 (11.4%)	28 (15.9%)	
G4	1 (0.6%)	1 (0.6%)	

### Immune infiltration analysis

The expression data (ESTIMATE) algorithm was used to estimate the number of stromal and immune cells in malignant tumor tissues and to calculate stromal score, immune score, and tumor purity [23]. A statistically significant difference was determined to use the “CIBERSORT” R package in analyzing infiltration of 22 immune cell types [24]. By applying the ‘ggplot2’ package, immune checkpoint analysis was plotted.

### TCGA database gene mutation analysis and drug sensitivity analysis

The TCGA database contains 175 samples for mutation detection, of which 156 (89.1%) are mapping samples. In order to analyze gene mutations, we divided PC patients into two groups based on their median BICC1 expression. Waterfall map of gene mutation was plotted by applying the ‘GenVisR’ package [25]. From Genomics of Drug Sensitivity in Cancer (GDSC) [26] and Genomics of Therapeutics Response Portal (CTRP) [27], the expression of BICC1 correlates linearly with small molecules using the Pearson correlation coefficient.

### Statistical methods

The Mann–Whitney test was used for categorical data, and Fisher’s exact test was used for continuous data. Clinicopathological characteristics and mRNA expression were also analyzed using chi-squared tests. As part of the analysis of survival curves, the log-rank test and Kaplan-Meier analysis were used. In addition, the Cox regression model was used to estimate the hazard ratios (HR) and 95% confidence intervals (CI). To test whether two continuously correlated variables are correlated, Pearson correlation analysis was conducted. Software version 4.0.2 of R Studio was used to perform all bioinformatics analyses. For all statistical analyzes, SPSS 22.0 (SPSS, USA) was used. *P* values < 0.05 were considered significant.

## Results

### The Discovery process of BICC1

**Fig. 1:**
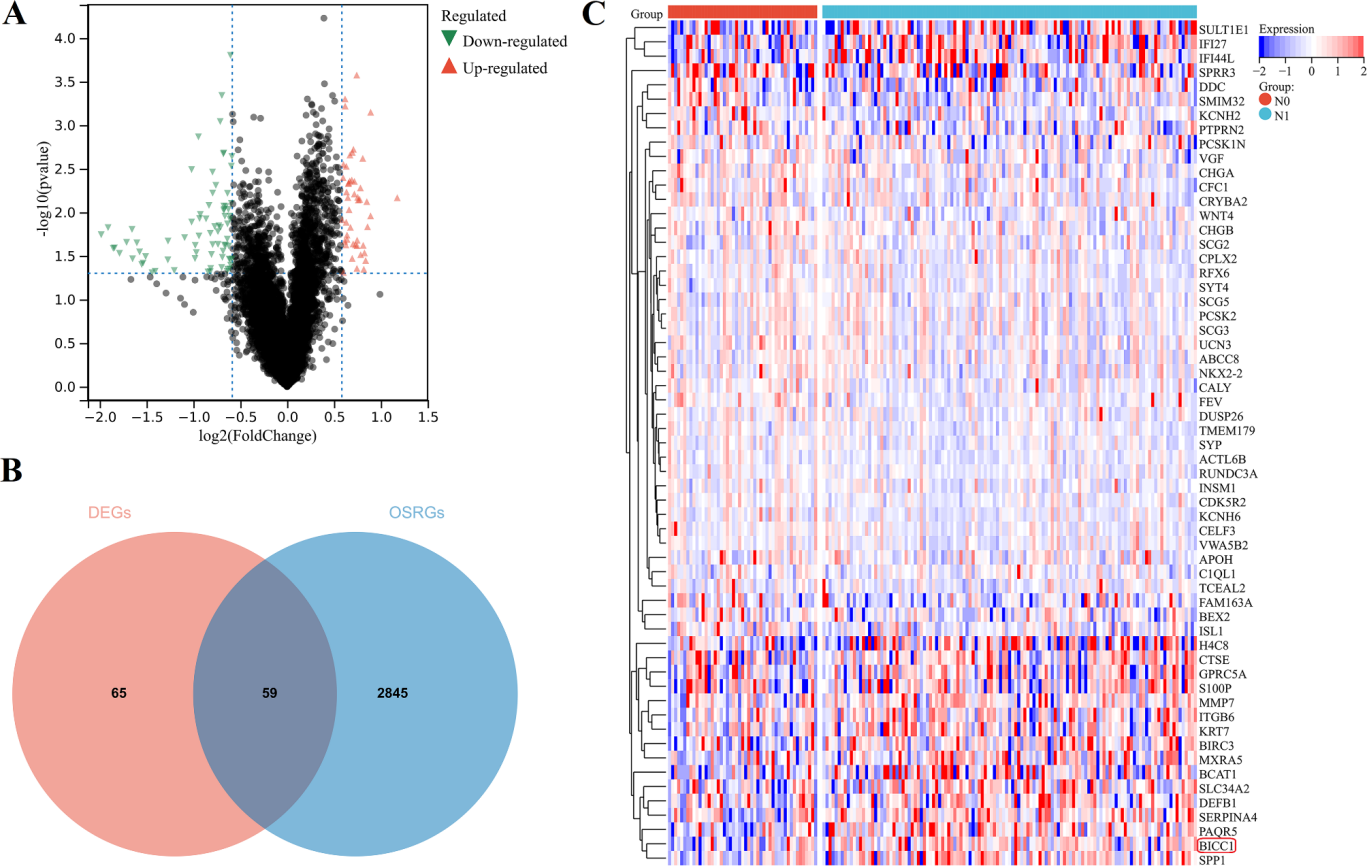
The discovery process of BICC1 in TCGA. **A** The DEGs according to N stage in TCGA. **B** Intersection of DEGs and OSRGs. **C** Heatmap illustrating the expression of the 59 genes. TCGA: The Cancer Genome Atlas; DEGs: Differentially Expressed Genes; OSRGS: Overall survival related genes

​As a first step, we divided TCGA patients into two groups according to N stages, including 48 patients with stage N0 disease and 123 patients with stage N1 disease. There were 124 DEGs identified, including 48 upregulated genes and 76 downregulated genes (Fig. 1A). Then, we identified a total of 2904 OSRGs based on univariate Cox regression analysis. We intersected DEGs and OSRGs to produce 59 candidate genes (Fig. 1B). BICC1, as an RNA binding protein, plays an important role in various physiological and pathological processes, but its biological function in PC progression has not yet been elucidated. So we chosed BICC1 as the target gene for this study. We found that BICC1 was significantly highly expressed in PC tissues with N1 stage. It was visualized through the heatmap (Fig. 1C).

### Expression level of BICC1 in PC

**Fig. 2:**
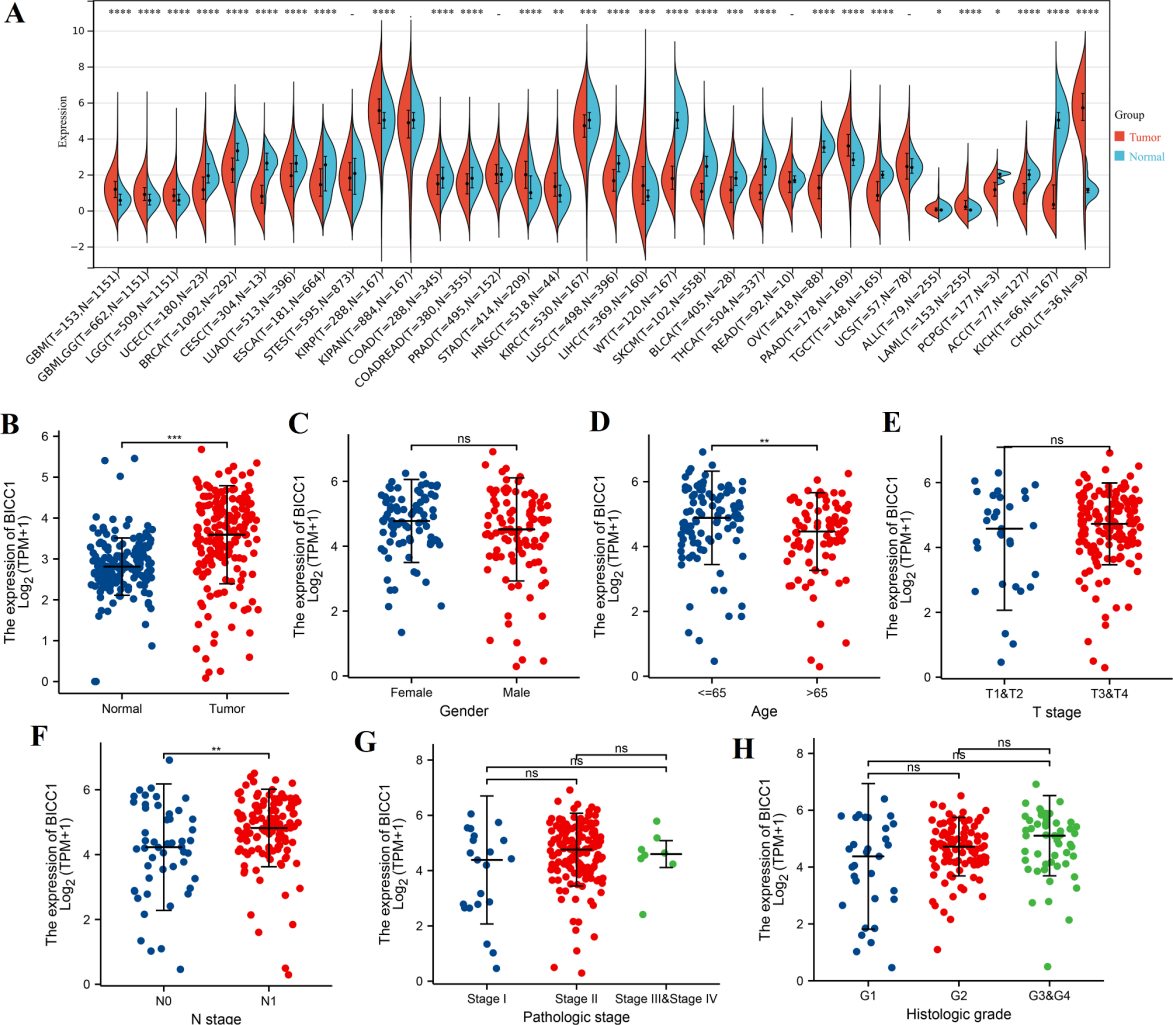
The expression of BICC1. **A** Differential expression levels of BICC1 in tumors versus normal tissues based on TCGA and GTEx databases. **B** The expression levels of BICC1 in tumors versus normal tissues based on TCGA. The association between the expression levels of BICC1 and clinical characteristics in PC. It showed that BICC1 expression remained elevated in different clinical subgroups of **C** gender; **D** age; **E** T stage; **F** N stage; **G** pathologic grade; **H** histologic grade. PC: pancreatic cancer. TCGA: The Cancer Genome Atlas; GTEx: Genotype-Tissue Expression; PC: Pancreatic cancer

By combining the TCGA and GTEx databases, we first observed BICC1 expression in different human cancers. The mRNA levels of BICC1 were increased in 10 cancers, such as pancreatic adenocarcinoma, glioblastoma multiforme, glioma, stomach adenocarcinoma, kidney renal papillary cell carcinoma, and cholangiocarcinoma. In addition, the mRNA levels of BICC1 were decreased in 18 cancers, such as breast invasive carcinoma, uterine corpus endometrial carcinoma, endocervical adenocarcinoma, and cervical squamous cell carcinoma (Fig. 2A). In the TCGA databases, In PC tissues, BICC1 expression was higher than in normal tissues (*P* < 0.001) (Fig. 2B). There was an increase in expression of BICC1 in PC tissues of different genders, age group, T stage, N stage, pathological stage, and histological grade (Fig. 2C-H). We found that the mRNA level of BICC1 was differentially expressed according to age and N stage (*P* < 0.01) (Fig. 2D F).

### Elevated expression of BICC1 protein in PC

**Fig. 3:**
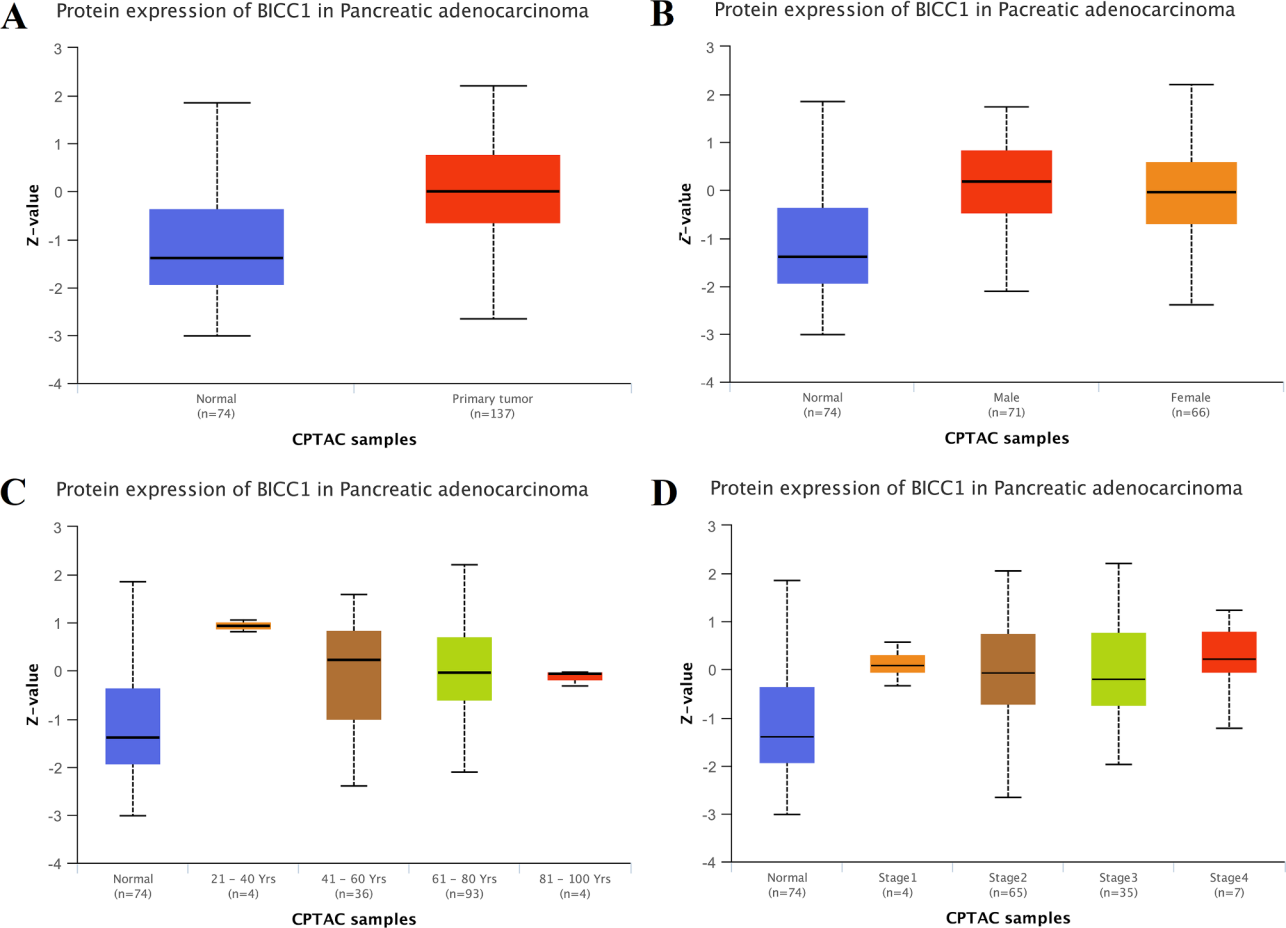
Results of protein level expression of BICC1 in UALCAN database. **A** Upregulation of BICC1 protein level expression in PC tissues. **B** BICC1 protein levels are elevated in different sexes than in normal samples. **C** BICC1 protein levels were elevated in different age subgroups compared to normal samples. **D** BICC1 protein levels were elevated in different pathologic grades compared to normal samples. UALCAN: University of Alabama at Birmingham Cancer; PC: Pancreatic cancer

BICC1 protein expression was further examined in PC tissues as part of our study. We observed a significant rise in the expression level of BICC1 protein in PC tumor tissues extracted from the UALCAN online tumor database (Fig. 3A). This behavior is seen in tumor samples from a variety of ages, sexes, and disease stages (Fig. 3B-D).

### Functional prediction of BICC1 in PC

**Fig. 4:**
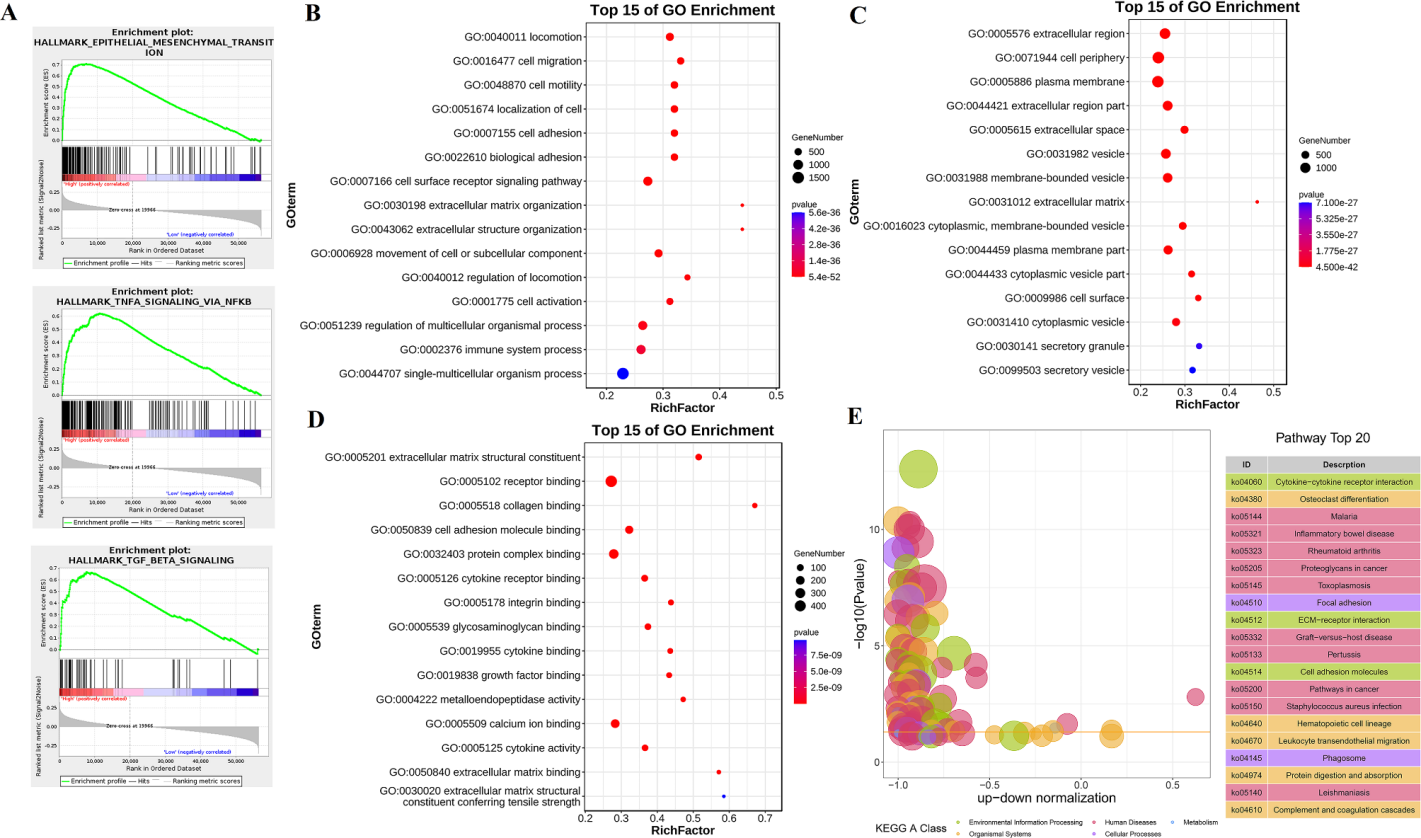
The role of BICC1 in PC. **A** GSEA enrichment in TCGA database. **B** GO enrichment analysis of Biological Process. **C** GO enrichment analysis of Cellular Component. **D** GO enrichment analysis of Molecular Function. **E** KEGG enrichment analysis. PC: Pancreatic cancer; TCGA: The Cancer Genome Atlas; GSEA: Gene Set Enrichment Analysis; GO: Gene Ontology; KEGG: Kyoto Encyclopedia of Genes and Genomes

To clarify the underlying mechanism of BICC1 in PC progression, the GSEA enrichment analysis was showed in view of TCGA groups with different BICC1 expression. The most enriched gene signature was the HALLMARK Epithelial mesenchymal transition (EMT) pathway (Fig. 4A). Moreover, the TNFα/NF-kB signaling pathway as well as the TGF-β signaling pathway were significantly enriched (Fig. 4A). Based on GO functional enrichment, BICC1 mainly affects biological processes (BP), including locomotion, cell migration, and cell motility (Fig. 4B); cellular components (CC) including plasma membrane, extracellular region, and cell periphery (Fig. 4C); moreover, molecular function (MF) including receptor binding, extracellular matrix structural constituent, and collagen binding (Fig. 4D). KEGG analysis found that BICC1 was associated with cytokine-cytokine receptor interaction, osteoclast differentiation, and ECM-receptor interaction (Fig. 4E).

### The role of BICC1 in evaluating prognosis

**Fig. 5:**
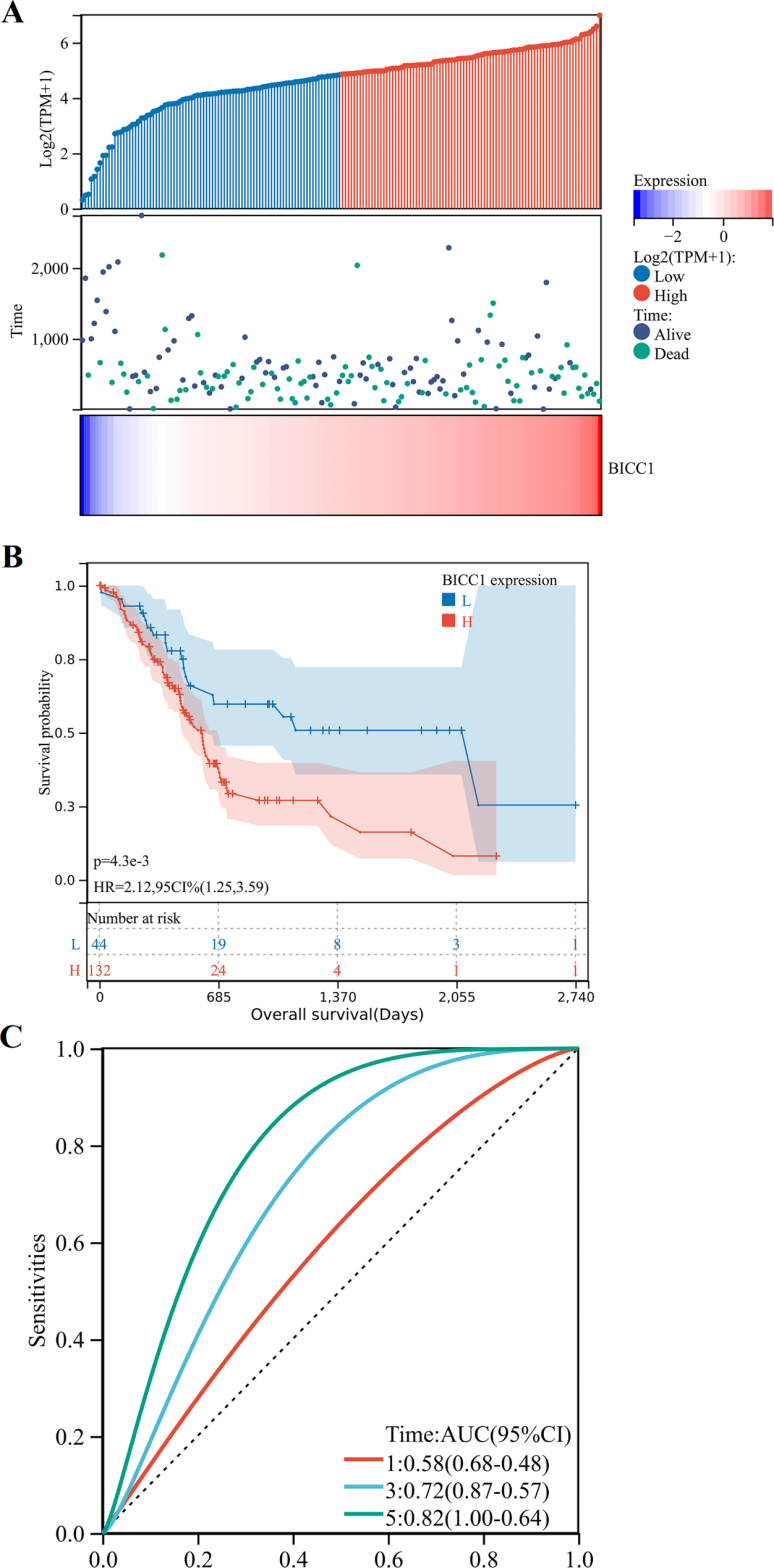
Prognostic analysis of BICC1 expression levels on overall survival of PC in TCGA dataset. **A** Heatmap of BICC1 expression distribution, survival status and BICC1 expression profile. **B** Kaplan–Meier analysis based on BICC1 expression. (**c**) Time-dependent ROC curve of BICC1 expression predicting prognostic risk of patients. PC: Pancreatic cancer; TCGA: The Cancer Genome Atlas; ROC: Receiver Operating Characteristic

**Fig. 6:**
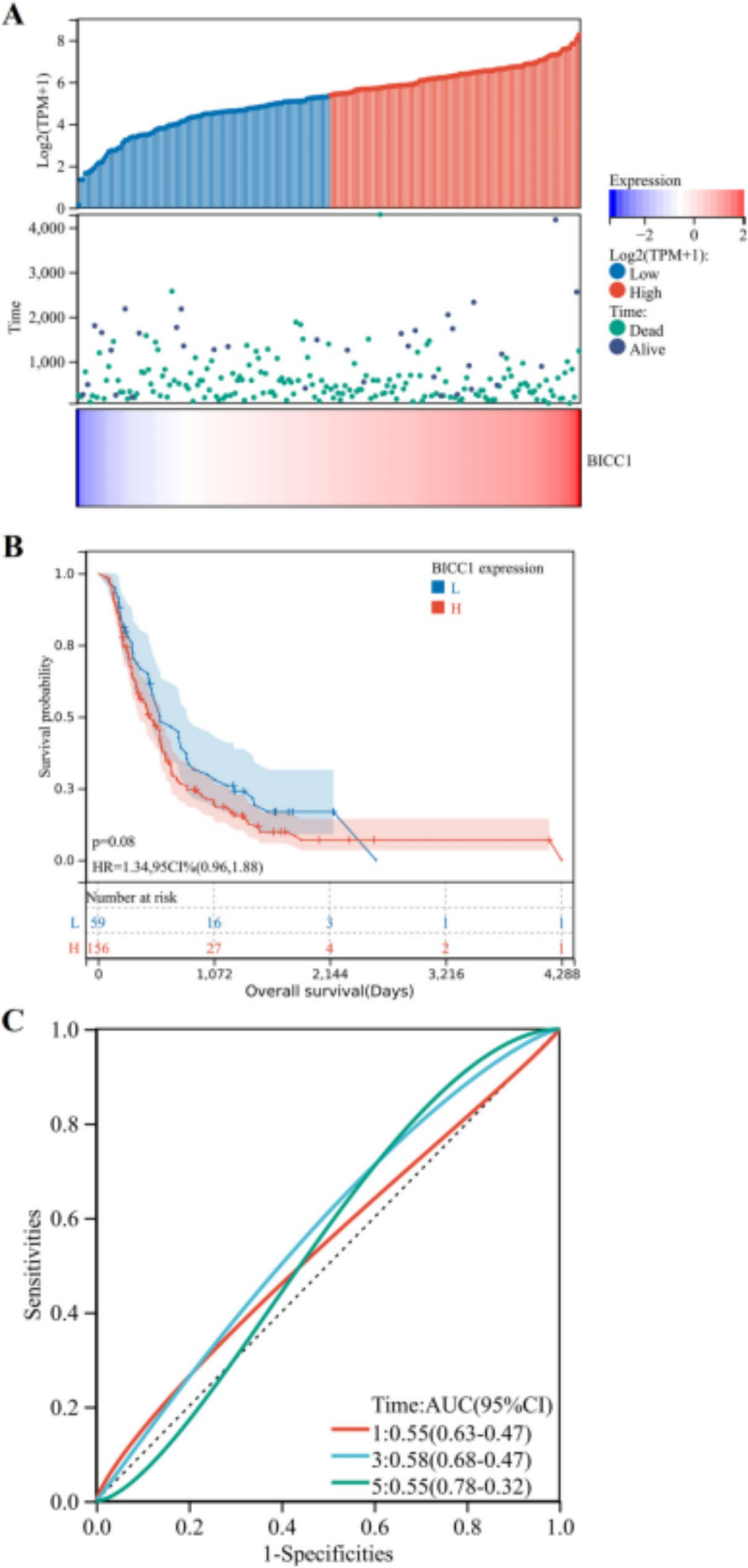
Prognostic analysis of BICC1 expression levels on overall survival of PC in ICGC dataset. **A** Heatmap of BICC1 expression distribution, survival status and BICC1 expression profile. **B** Kaplan–Meier analysis based on BICC1 expression. (**c**) Time-dependent ROC curve of BICC1 expression predicting prognostic risk of patients. PC: Pancreatic cancer; ICGC: International Cancer Genome Consortium; ROC: Receiver Operating Characteristic

Utilizing the TCGA and ICGC data bases, we investigated whether BICC1 expression and patient survival were correlated in PC patients. In order to differentiate between patients with high and low BICC1 expression, we divided the patient records by median BICC1 expression (Fig. 5A). A Kaplan-Meier survival curve showed that high BICC1 expression was associated with poor prognosis in PC (*P* = 0.0043) (Fig. 5B). We additionally plot the time-dependent Receiver Operating Characteristic (ROC) curves for BICC1 prediction of survival in PC patients. ROC curves with time-dependent AUC values at 5 years were 0.82 (Fig. 5C). By using the ICGC database, we validated the results (Fig. 6A). There was an association between poorer OS and high BICC1 expression, but there is no significant statistical significance (*P* = 0.08) (Fig. 6B). ROC curves with time-dependent AUC values at 5 years were lower than 0.6 (Fig. 6C). Consistent findings like these point to BICC1’s potential as a biological marker for determining PC patients’ prognoses.

### A prognostic model based on BICC1 expression to predict the prognosis of PC patients

**Fig. 7:**
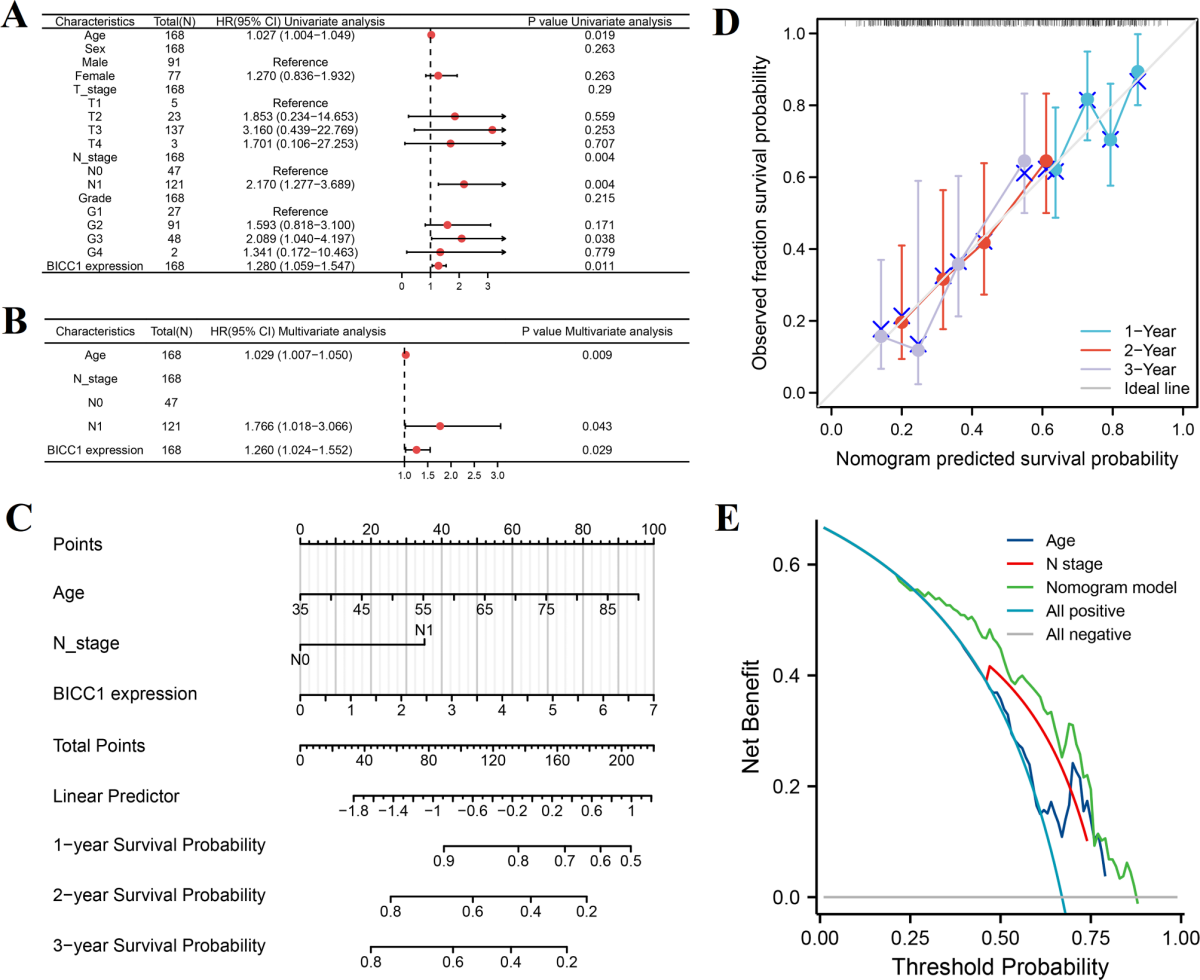
Prognostic risk model for PC was constructed in TCGA database. **A** Uunivariate Cox regression analysis. **B** Multi-Cox regression analysis. **C** Nomogram that can predict the 1-, 2-, and 3-year survival probability of PC. **D** Calibration curve of the prognostic risk model for PC. **E** Decision curve analysis of the prognostic risk model for PC. PC: Pancreatic cancer

We considered that not only the expression of BICC1 affects the prognosis of PC patients. A variety of clinical characteristics were considered in this study, including age, gender, T stage, N stage, and histological grade. The results showed that the expression of BICC1 is closely related to the prognosis of PC patients based on the forest plot produced after univariate Cox regression analysis (Fig. 7A). BICC1 expression (*P* = 0.029), age (*P* = 0.009), and N stage (*P* = 0.043) were identified as prognostic factors in the multivariate Cox proportional risk regression analysis (Fig. 7B). Then, we combined expression of BICC1, age and N stage for constructing the OS nomogram (Fig. 7C). Based on the nomogram calibration curve, the prediction results of this model were highly consistent with all patient observations (Fig. 7D). Decision curve analysis showed that the prognostic model was better than age and N stage alone at 5 years [0.616 (0.584–0.648) vs. 0.563 (0.531–0.595) vs. 0.563 (0.537–0.589)] (Fig. 7E).

### Correlation between BICC1 expression and immune characteristics in PC

**Fig. 8:**
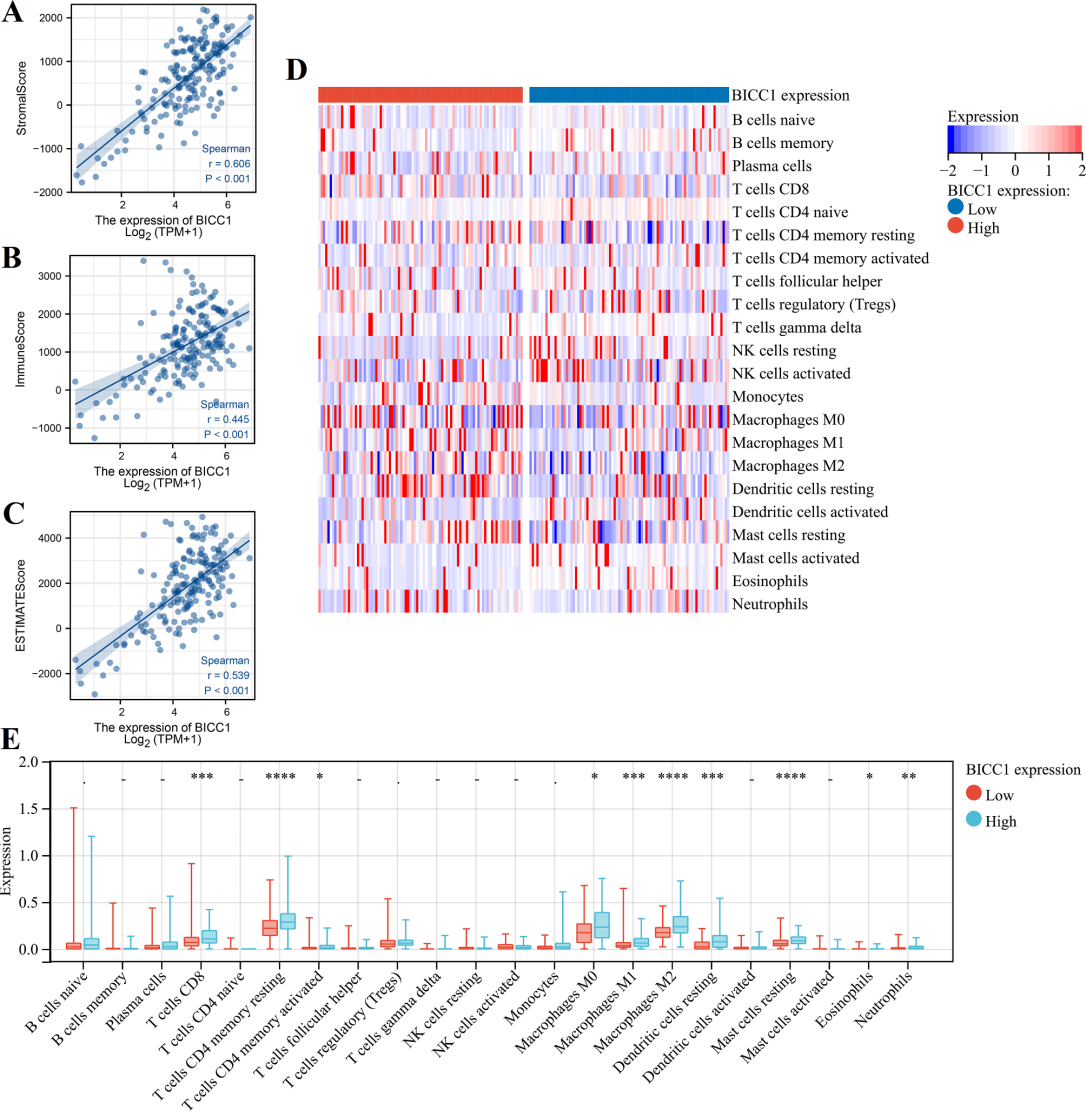
Immune infiltration analysis of BICC1 in PC. **A-C** BICC1 expression correlated with stomal score, immune score and ESTIMATE score calculated by ESTIMATE algorithm. **D, E** 22 immune related cells evaluated by CIBERSORT algorithm between different BICC1 expression. PC: Pancreatic cancer

**Fig. 9:**
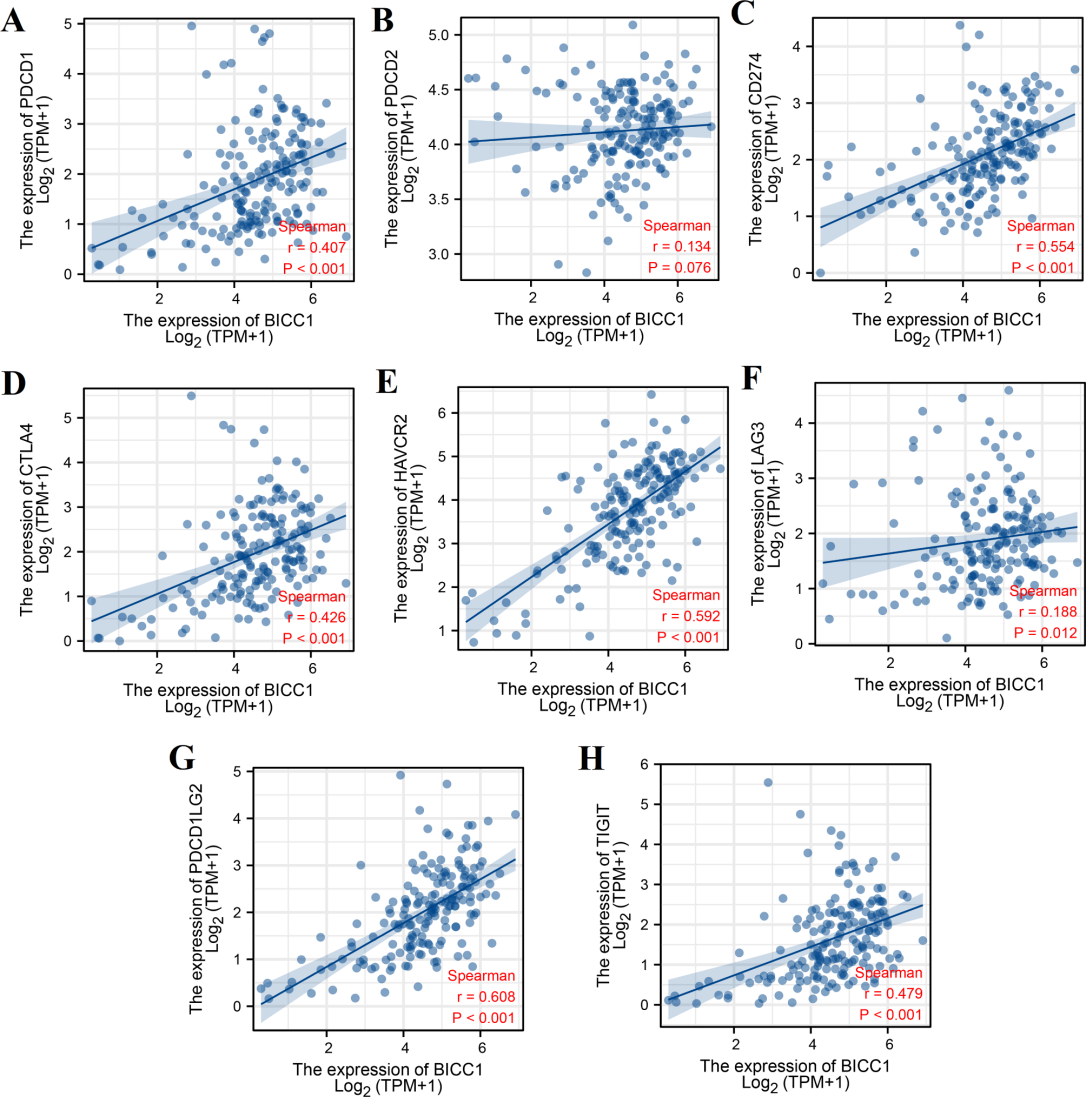
Correlation of BICC1 expression with immune checkpoint expression in PC. **A** The correlation of BICC1 expression with PDCD1 expression. **B** The correlation of BICC1 expression with PDCD2 expression. **C** The correlation of BICC1 expression with CD247 expression. **D** The correlation of BICC1 expression with CTLA-4 expression. **E** The correlation of BICC1 expression with HAVCR2 expression. **F** The correlation of BICC1 expression with LAG3 expression. **G** The correlation of BICC1 expression with PDCD1LG2 expression. **H** The correlation of BICC1 expression with TIGIT expression. PC: Pancreatic cancer

We analyzed the correlation between BICC1 expression and immune cell infiltration. ESTIMATE algorithm revealed a positive correlation between BICC1 expression and stromal score (r = 0.606, *P* < 0.001), immune score (r = 0.445, *P* < 0.001), and ESTIMATE score (r = 0.539, *P* < 0.001) in the TCGA dataset (Fig. 8A–C). Moreover, CIBERSORT algorithm showed that BICC1 was positively correlated with CD8 T cells, memory CD4 T cells, M0 macrophage, M1 macrophage, M2 macrophage, resting dentritic cells, and resting mast cells (Fig. 8D, E). BICC1 expression was also significantly positively associated with multiple immunotherapeutic targets, including PDCD1, PDCD2, CD247, CTLA-4, HAVCR2, LAG3, PDCD1LG2 and TIGIT (Fig. 9A-H).

### Genetic alteration and drug sensitivity analysis of BICC1

**Fig. 10:**
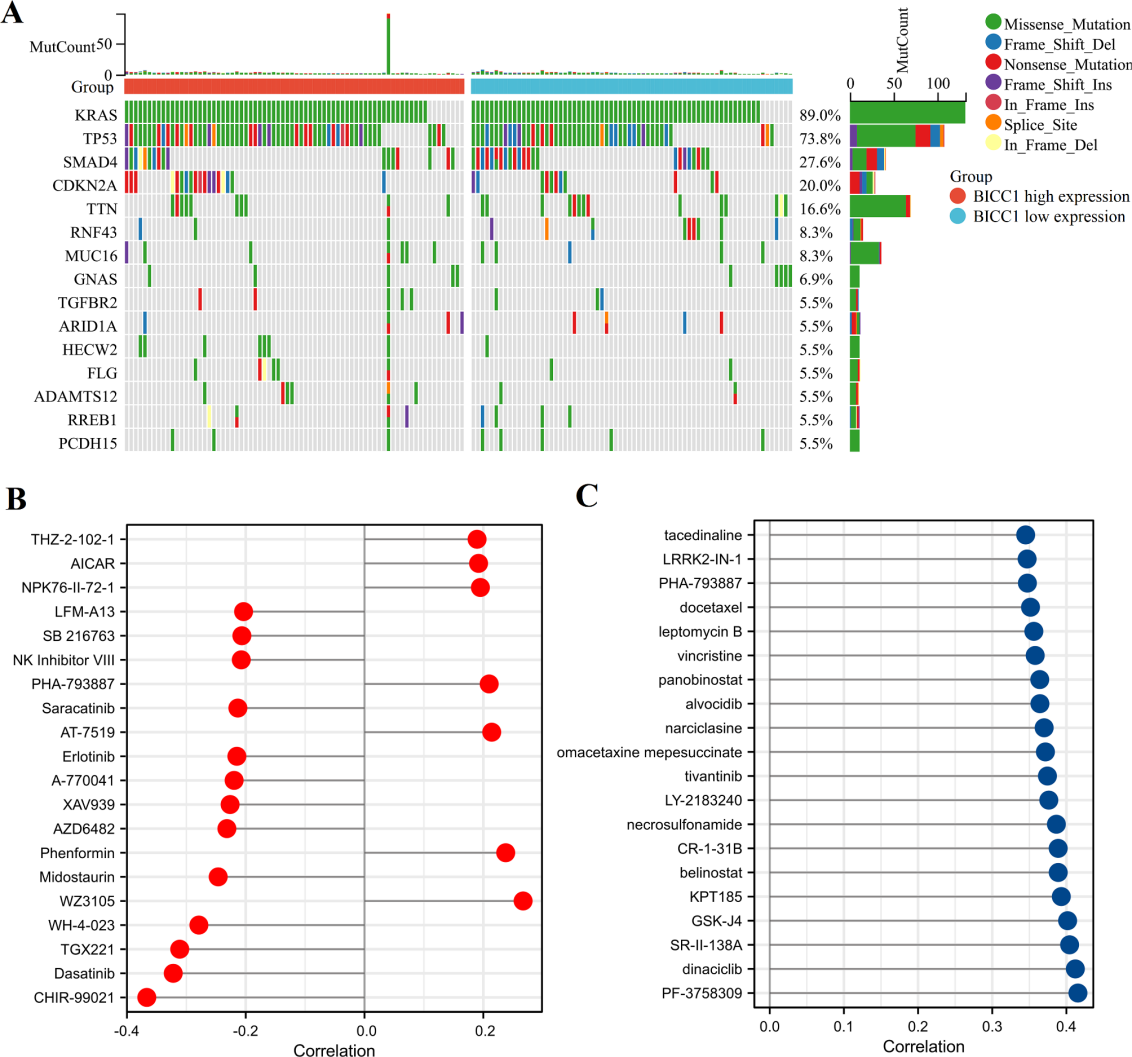
Genetic alteration and drug sensitivity analysis of BICC1. **A** Genetic alterations in different BICC1 expression groups from the TCGA database. **B** Drug-sensitivity analysis of BICC1 in Genomics of Drug Sensitivity in Cancer (GDSC); **C** Drug-sensitivity analysis of BICC1 in Genomics of Therapeutics Response Portal (CTRP). PC: Pancreatic cancer; TCGA: The Cancer Genome Atlas

According to gene mutation data in the TCGA database, we found that the genetic alterations mainly included missense mutations. KRAS, TP53, and SMAD4 were the top three most commonly altered genes in the PC. In addition, TP53 gene mutation was more frequent in PC patients with high BICC1 expression; SMAD4 gene mutation was more frequent in PC patients with low BICC1 expression (Fig. 10A). Genetic alterations are potential targets for antitumor drug matching, and influence clinical treatment intervention. We analyzed the differences between the different BICC1 expression for sensitivity to different antitumor drugs. BICC1 was more sensitive to WA3105, phenformin, AT7519, PHA-793,887, and NRK76-II-72-1 in the GDSC database (Fig. 10B). Moreover, it was also sensitive to PF-3,758,309, Dinaciclib, SR-II-138 A, GSK-J4, and KPT185 in the CTRP database (Fig. 10 C).

## Discussion

It is routine to assess prognosis and guide postoperative treatment based on tumor invasion, regional lymph nodes, and distant metastases in PC [28]. A few studies retrospectively analyzed the significance of the number of positive lymph node metastases in evaluating the prognosis of PC [29, 30]. However, there is considerable heterogeneity in the evaluation of postoperative N staging due to the skill of surgeons and the experience of pathologists. Therefore, it is desirable to screen for reliable biomarkers based on differences in lymph node metastasis to assess the prognosis and biological function of PC. Through bioinformatics, BICC1 was found to be relevant to lymph node metastasis in PC patients. In this work, we investigated the mRNA and protein expression of BICC1, which is significantly expressed in tumor tissue, in PC patients. BICC1 expression, age, and N stage were independent predictors of OS in PC patients based on our analysis of clinical characteristics and prognosis. Finally, BICC1 expression and clinicopathological characteristics were used to develop a prognostic model for PC. PC patients with high BICC1 expression had a poor prognosis, indicating that BICC1 is a prognostic factor.

​BICC1 encodes an RNA binding protein whose primary role is to mediate the maturation, transport, localization, and translation of RNA [31, 32]. BICC1 is widely expressed in various human tissues, particularly in the kidney, and plays a role in regulating vertebrate embryogenesis [12, 33]. The biological behaviors of cell proliferation and apoptosis are regulated by BICC1, which has been linked to the occurrence and progression of tumors [14, 34, 35]. Further, BICC1 is associated with PC immune cell infiltration [12]. However, the specific function of BICC1 in tumor progression is still debated. So far, only Wang et al. [14] had probed into the intention of BICC1 in cancer. The findings suggest that tumor cells are stimulated by BICC1 by inhibiting apoptosis, which leads to a lower survival rate for people with oral cancer. The expression of BICC1 was irregular in varied tumor types. However, no studies have confirmed the prognostic value of BICC1 in PC. Moreover, we discovered that increased BICC1 expression was evidently relevant to the N stage of PC. This indicates that it may be a possible prognostic biomarker.

​Further exploration verify BICC1 as a effective biomarker, gene enrichment analysis found that BICC1 was closely related to the EMT pathway and cell migration through GSEA and GO enrichment analyses. In the development of EMT, cell-cell, TGF-β pathways, and cell-extracellular matrix interactions are remodeled, which results in epithelial cell separation from each other and basement membrane separation and activates different transcription procedures to facilitate the outcome of the interstitium [36]. During tumor occurrence and development, EMT endows cancer cells with increased tumor initiation and metastasis potential, and increases cancer cells’ drug tolerance [36]. The strong correlation between BICC1 and the EMT pathway demonstrates its great potential as a biomarker and suggests new ideas for treating PC with BICC1.

In addition, the interaction and close relationship between stromal cells and immune cells in tumor microenvironment also regulate tumor progression by influencing the EMT pathway. This study confirmed that BICC1 expression was significantly correlated with tumor stroma by the ESTIMATE algorithm. The biological behavior of cancer cells to generate EMT will be regulated by a large number of growth factors and cytokines including TGF-β, IL-6, EGF, VEGF and HGF, secreted from Cancer-associated fibroblasts (CAFs) [37, 38]. Using the CIBERSORT algorithm, the strong correlation between 22 immune cells and BICC1 expression were presented. It was shown that BICC1 overexpression is associated with stronger T cell and macrophage infiltration. The study of Goebel et al. [39] found that pancreatic ductal epithelial cells co-cultured in vitro with T cells lose expression of E-cadherin and acquire a spindle-shaped mesenchymal morphology. Recent studies also shown that mesenchymal carcinoma cells induce the formation of tumor-associated macrophages (TAMs) which secreting by GM-CSF. The recruited TAMs further secrete reciprocating CCL18 to induce EMT and promote breast cancer metastasis [40]. These findings furthermore confirm that BICC1 influences immune invasion in the tumor microenvironment through the EMT pathway to promote PC progression.

​In terms of treatment, we analyzed the sensitivity of BICC1 to different anti-tumor drugs and found that the sensitivity or resistance of a large number of chemotherapy drugs or targeted drugs was related to the expression of BICC1. This raised the prospect that PC patients with elevated BICC1 expression may respond better to these antitumor drugs. As a result, better clinical treatment interventions can also be developed for PC patients. Immunocheck point inhibitors have received increasing attention in the treatment of cancer, and have shown controllable safety and great efficacy [41]. In the future, the focus of PC therapy will shift from tumor cells to the tumor microenvironment. The tumor microenvironment will identify and ultimately kill cancer cells by mobilizing immune cells. We found that BICC1 expression was synergistic with a number of immune checkpoints that have been widely used in the clinic. Our study sheds new light on checkpoint inhibitors for future research in PC immunotherapy.

There are some limitations in our study. First, the TCGA database has a lot of limitations, and the BICC1 prognosis values for PC do not perform well in the ICGC database. More independent cohorts should be performed to verify our results. Second, in order to fully understand the precise molecular processes by which BICC1 promotes PC development, further in vitro and in vivo studies are required.

## Data Availability

The corresponding author can provide the data and R script used in this study upon reasonable request. All authors read and approved the final manuscript. We analyzed publicly available datasets in this study. These are available on The Cancer Genome Atlas (https://portal.gdc.cancer.gov/).
